# Chemical Constituents, Pharmacologic Properties, and Clinical Applications of *Bletilla striata*

**DOI:** 10.3389/fphar.2019.01168

**Published:** 2019-11-01

**Authors:** Delin Xu, Yinchi Pan, Jishuang Chen

**Affiliations:** ^1^Department of Cell Biology, Zunyi Medical University, Zunyi, China; ^2^Key Laboratory of Basic Pharmacology and Joint International Research Laboratory of Ethnomedicine of Ministry of Education, Zunyi Medical University, Zunyi, China

**Keywords:** *Bletilla striata*, chemical constituents, pharmacological activities, clinical application, quality control, toxicology

## Abstract

*Bletilla striata* is a plant from the Orchidaceae family that has been employed as a traditional Chinese medicine (TCM) for thousands of years in China. Here, we briefly review the published studies of the last 30 years that were related to chemical constituents, pharmacologic activities, and clinical applications of *B. striata*. Approximately 158 compounds have been extracted from *B. striata* tubers with clarified molecular structures that were classified as glucosides, bibenzyls, phenanthrenes, quinones, biphenanthrenes, dihydrophenanthrenes, anthocyanins, steroids, triterpenoids, and phenolic acids. These chemicals support the pharmacological properties of hemostasis and wound healing, and also exhibit anti-oxidation, anti-cancer, anti-viral, and anti-bacterial activities. Additionally, various clinical trials conducted on *B. striata* have demonstrated its marked activities as an embolizing and mucosa-protective agent, and its application for use in novel biomaterials, quality control, and toxicology. It also has been widely used as a constituent of many preparations in TCM formulations, but because there are insufficient studies on its clinical properties, its efficacy and safety cannot be established from a scientific point of view. We hope that this review will provide reference for further research and development of this unique plant.

## Introduction

*Bletilla striata* (Thunb.) Rchb. f. (Orchidaceae), also known as Bletillae Rhizoma, is considered to be merely an ornamental plant in Europe and the USA. However, *B. striata*, which is widely distributed in China, Japan, Korea, Mongolia, and Myanmar, is important because of its use in traditional Chinese medicine (TCM).

According to the earliest pharmacopeia of TCM, *Shennong’s Materia Medica Classic*, Chinese scholars were the first to describe the morphologic features and medicinal value of *B. striata* (Hossain, 2011; Chen et al., 2018). Other Chinese pharmacopeias have recorded the effect of its astringency upon hemostasis and analgesia, as well as its use for treating traumatic bleeding, ulcers, and swelling and chapped skin (He et al., 2016). Physicians in Korea and Japan have used *B. striata* to treat tuberculosis, whooping cough, bleeding of the stomach/duodenal ulcers, abscesses, swellings, and parasitic diseases (Rhee et al., 1982). Several studies have revealed its uses in TCM, its chemical constituents, and medicinal properties (Yang et al., 2019).

This review summarizes current knowledge regarding the chemical composition, bioactivities, and pharmacologic effects of *B. striata*. We also provide a brief summary to provide insights into the TCM-based uses of *B. striata,* and a scientific basis for developing new medicines utilizing this interesting plant.

## Chemical Constituents

**Figure 1:**
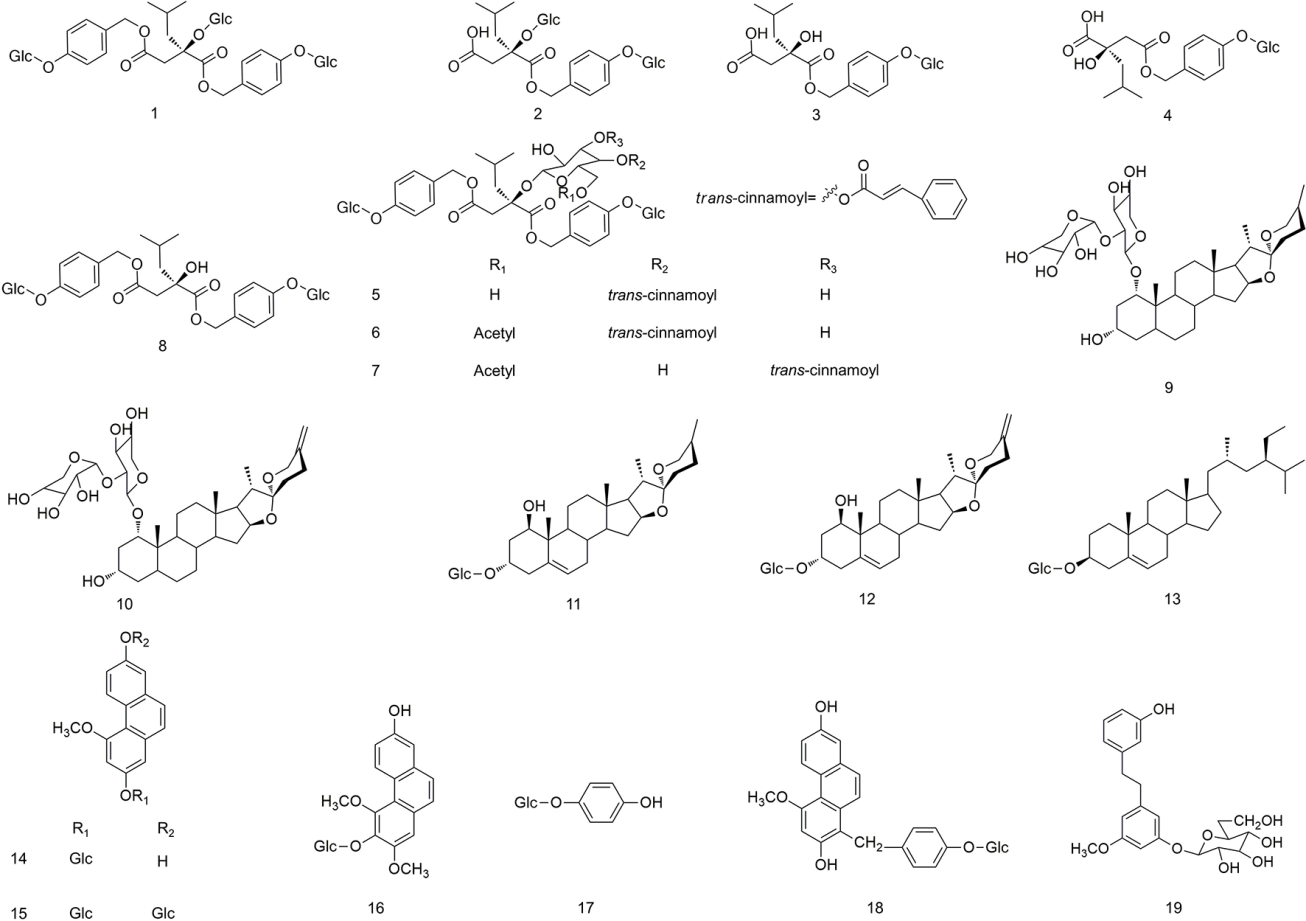
Chemical structures of glycoside compounds (**1–19**) isolated from *B. striata*.

**Figure 2:**
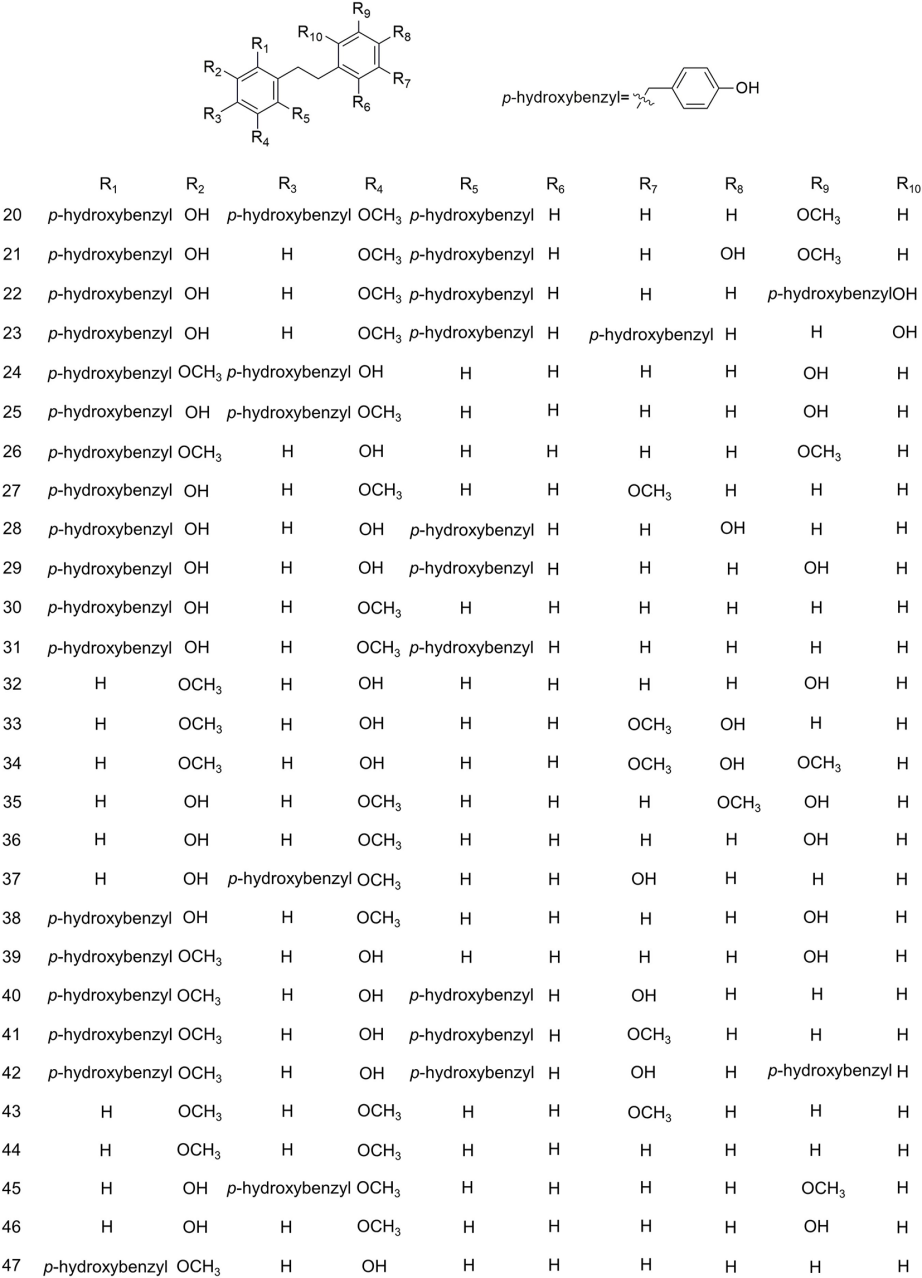
Chemical structures of bibenzyls (**20–47**) isolated from *B. striata*.

**Figure 3:**
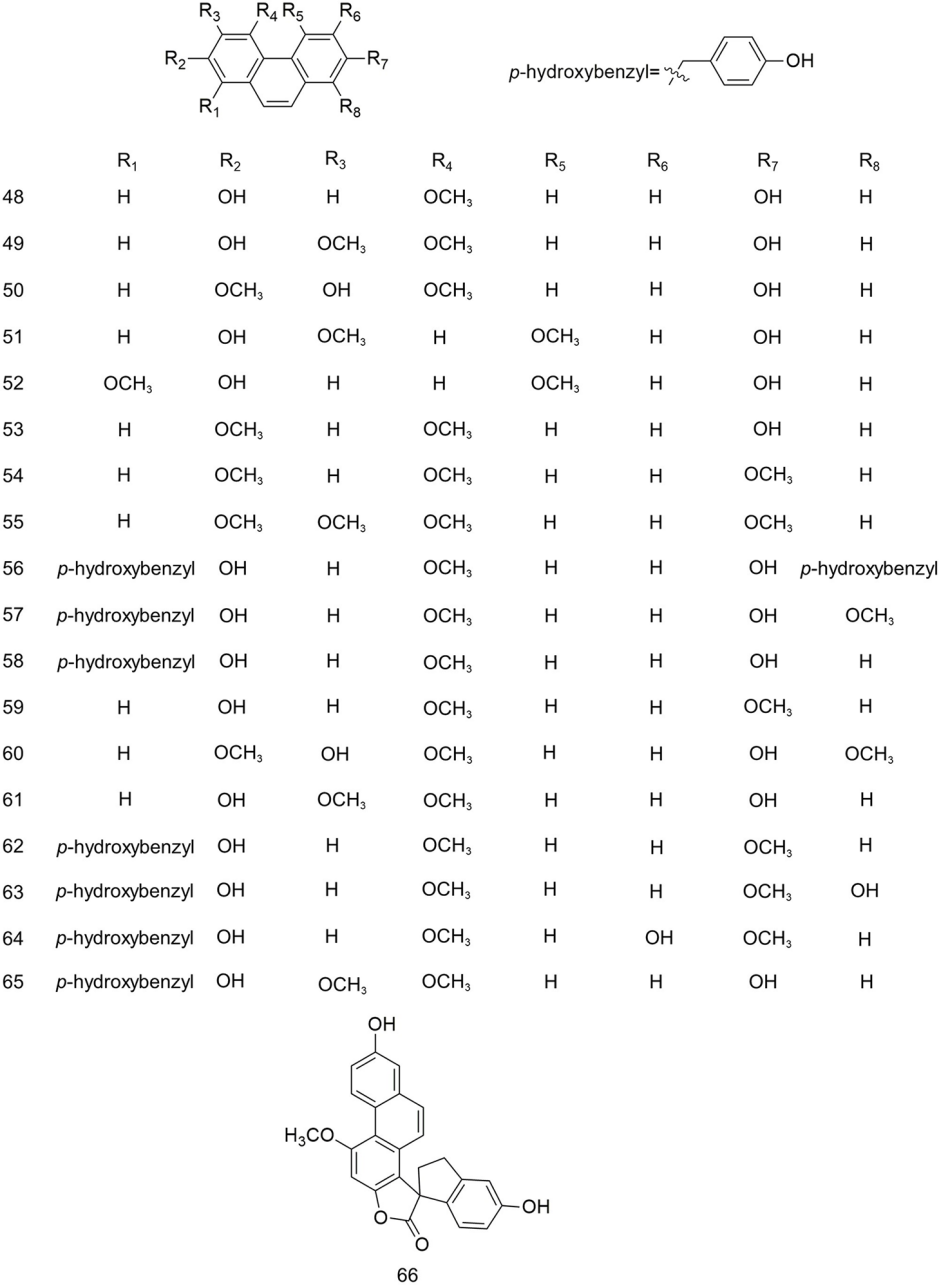
Chemical structures of phenanthrenes (**48–66**) isolated from *B. striata*.

**Figure 4:**
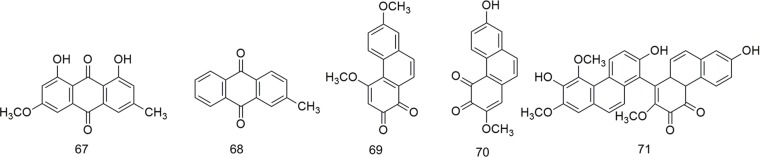
Chemical structures of quinones (**67–71**) isolated from *B. striata*.

**Figure 5:**
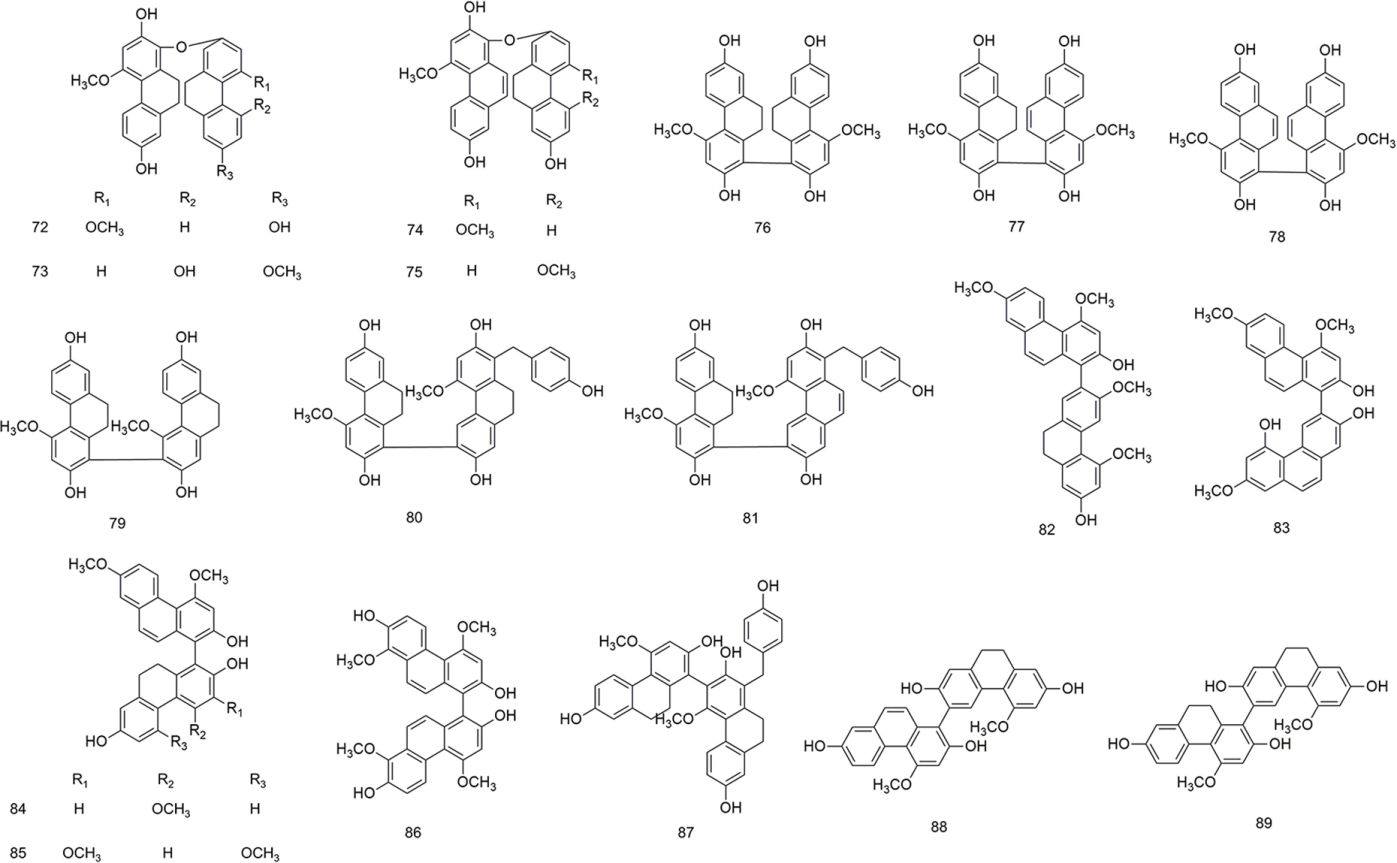
Chemical structures of biphenanthrenes (**72–89**) isolated from *B. striata*.

**Figure 6:**
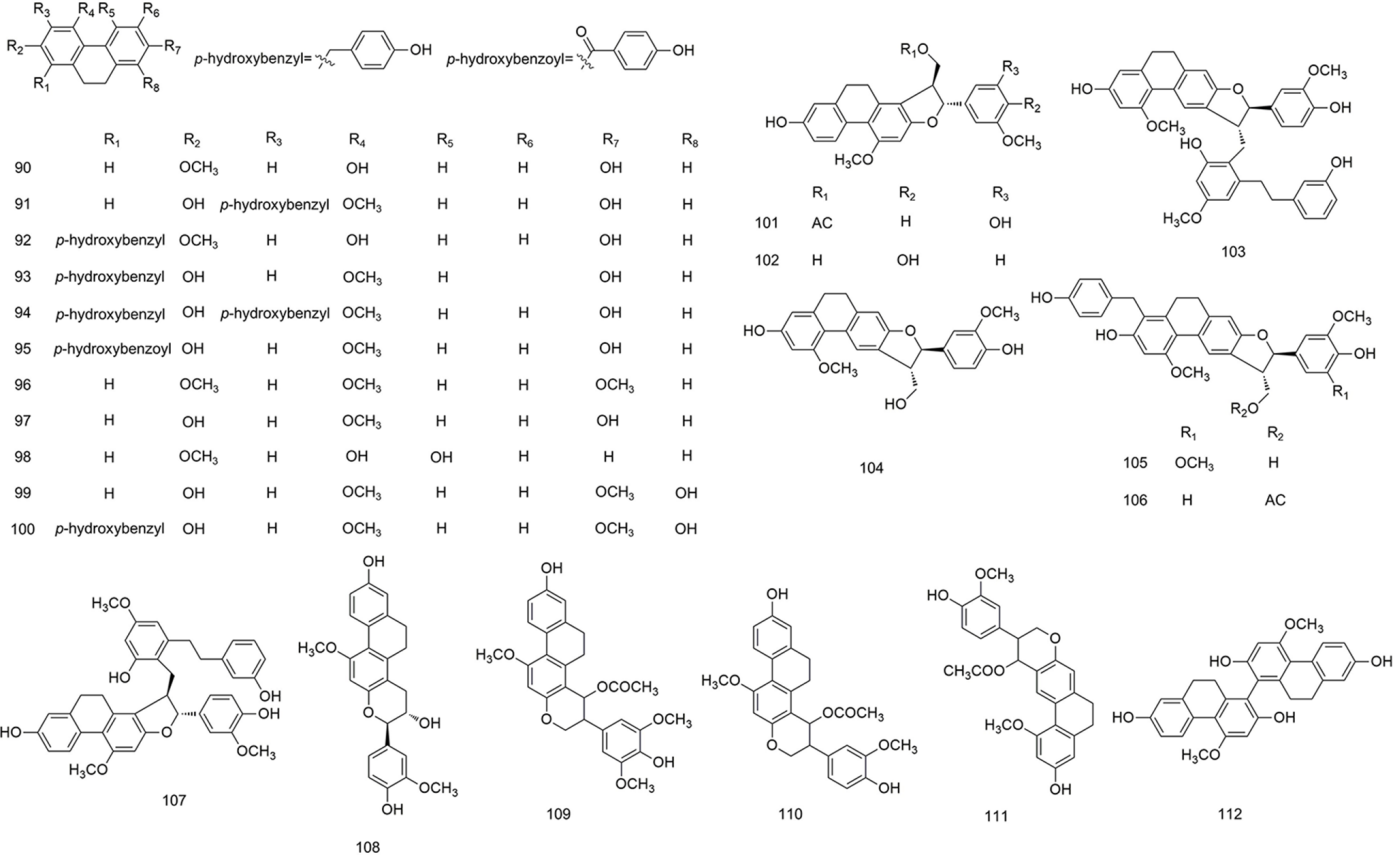
Chemical structures of dihydrophenanthrenes (**90–112**) isolated from *B. striata*.

**Figure 7:**
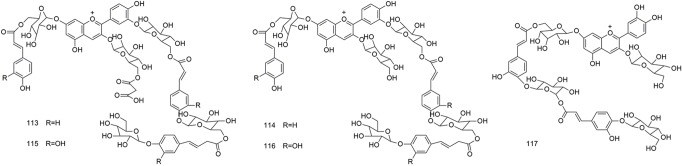
Chemical structures of anthocyanins (**113–117**) isolated from *B. striata*.

**Figure 8:**
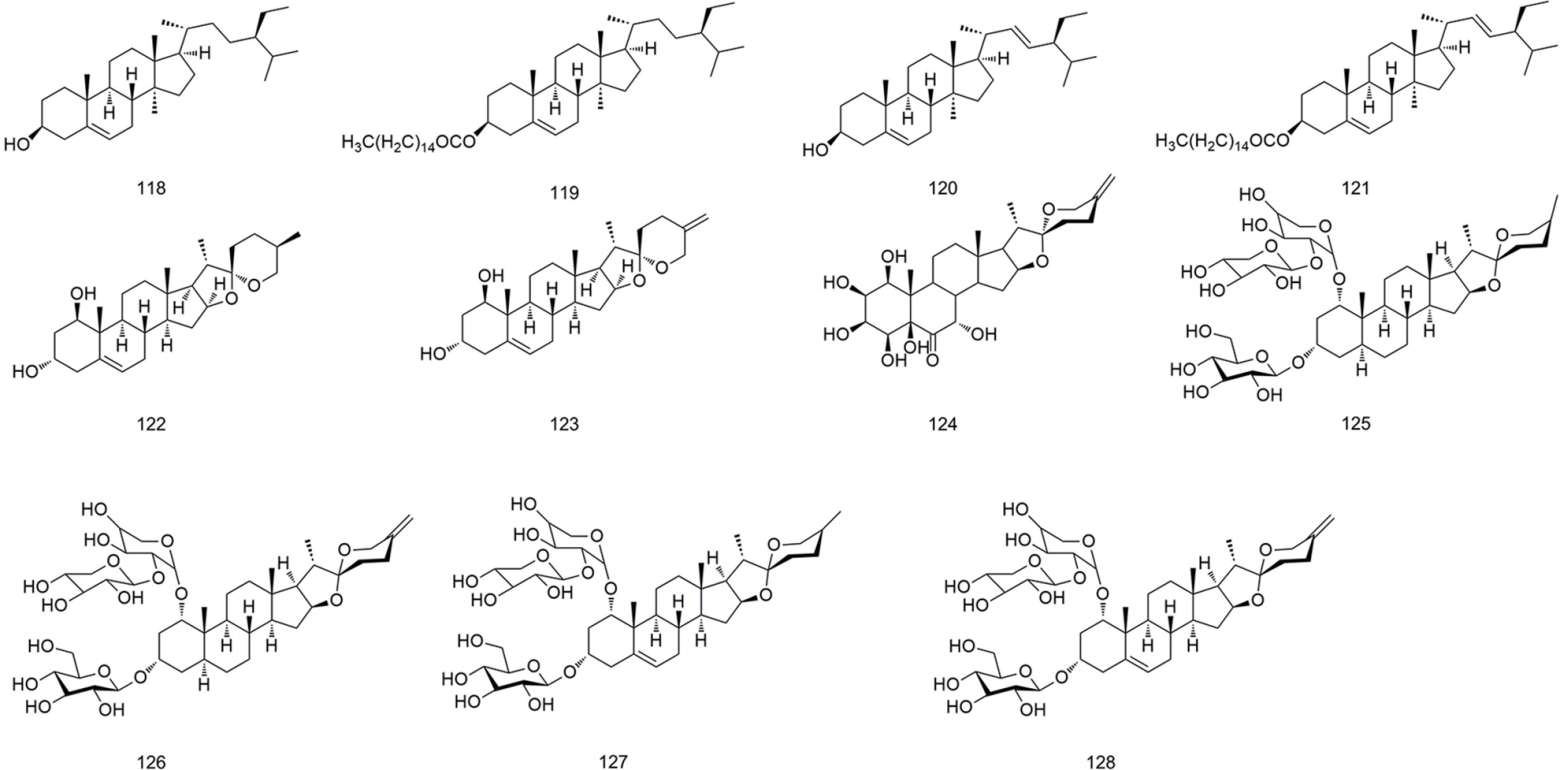
Chemical structures of steroids (**118–128**) isolated from *B. striata*.

**Figure 9:**
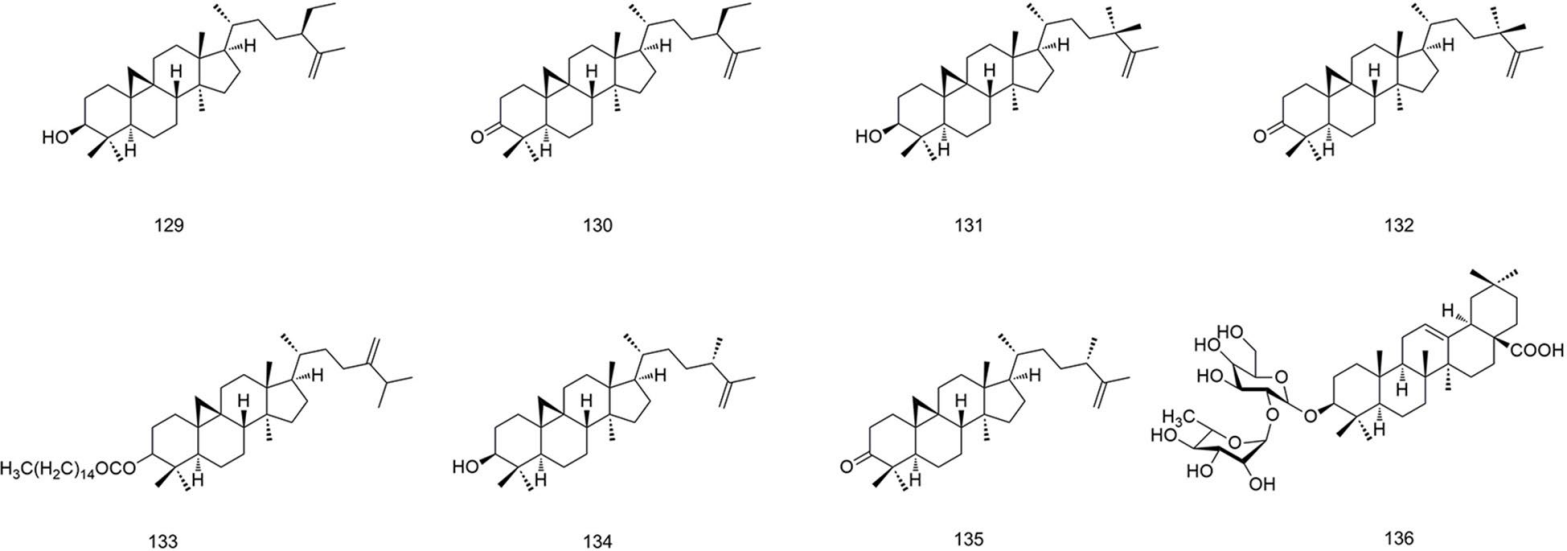
Chemical structures of triterpenoids (**129–136**) isolated from *B. striata*.

**Figure 10:**
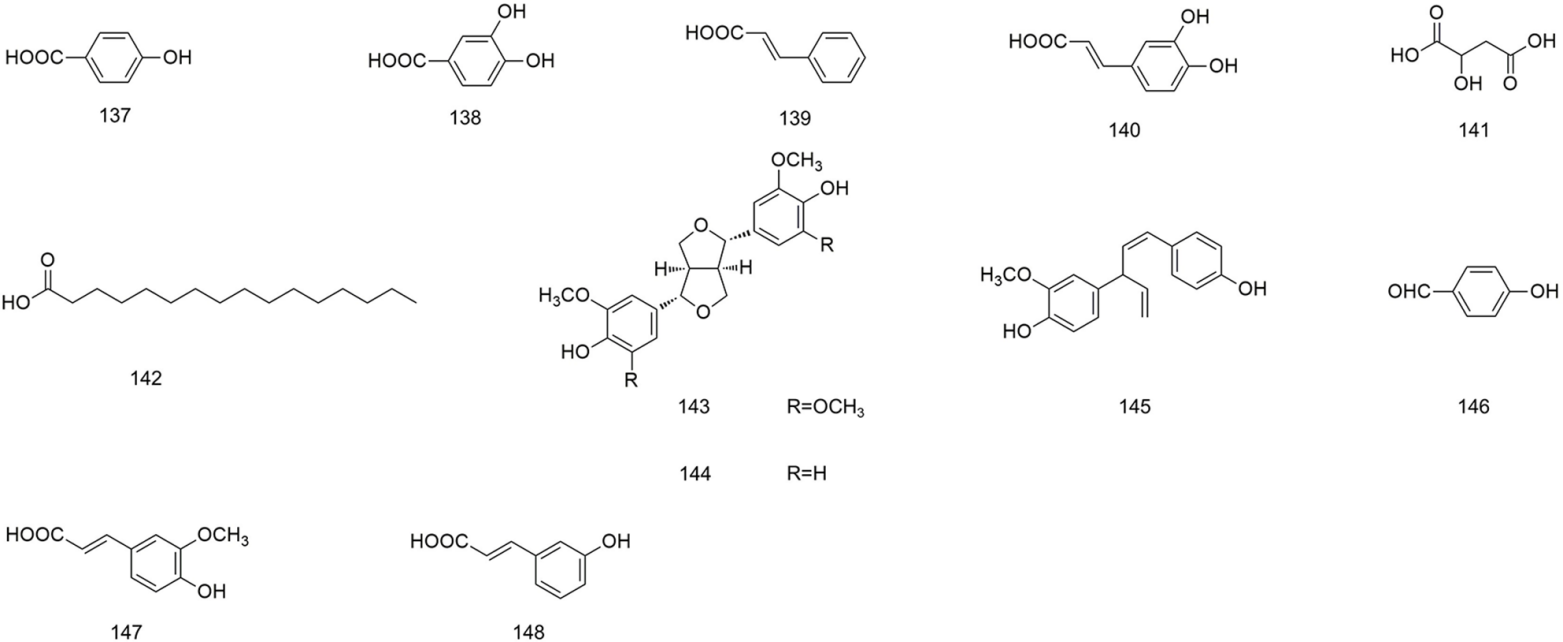
Chemical structures of phenolic acids (**137–148**) isolated from *B. striata*.

**Figure 11:**
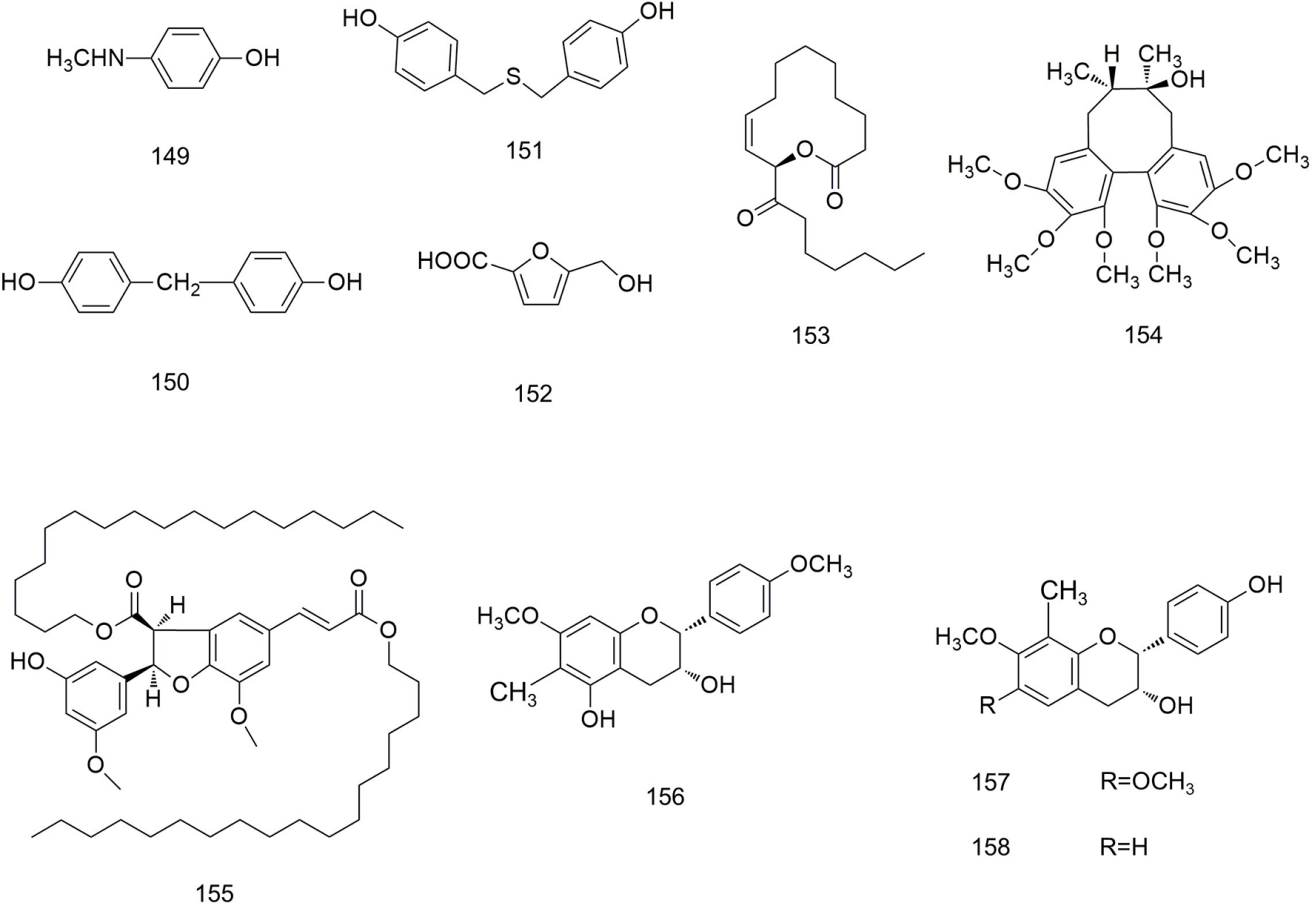
Chemical structures of other compounds (**149–158**) isolated from *B. striata*.

The study of the chemical constituents of *B. striata* can be traced back to the late 19th century, but Japanese scholars began systematic research only in 1983 (Takagi et al., 1983). Studies have shown that the chemical constituents of *B. striata* are mainly glucosides, bibenzyls, phenanthrenes, quinones, biphenanthrenes, dihydrophenanthrenes, anthocyanins, steroids, triterpenoids, and phenolic acids. Their names (**1–158**) appear in **Table 1**, and their structures (**1–158**) are shown in **Figures 1**–**11**. These chemical components form the material basis for the medicinal value of *B. striata*.

**Table 1:** List of 158 compounds isolated from *B. striata*.

No.	Compound Name	Chemical Formula	Plant Part	References
***Glycosides***				
1	dactylorhin A	C_40_H_56_O_22_	tubers	(Feng et al., 2008)
3	gymnoside I	C_21_H_30_O_11_	tubers	(Feng et al., 2008)
4	gymnoside II	C_21_H_30_O_11_	tubers	(Feng et al., 2008)
5	gymnoside V	C_49_H_62_O_23_	tubers	(Yan et al., 2014)
6	gymnoside IX	C_51_H_64_O_24_	tubers	(Yan et al., 2014)
7	gymnoside X	C_51_H_64_O_24_	tubers	(Yan et al., 2014)
8	militarine	C_34_H_46_O_17_	tubers	(Han et al., 2002b)
9	bletilnoside A	C_38_H_62_O_12_	roots	(Park et al., 2014)
10	bletilnoside B	C_38_H_60_O_12_	roots	(Park et al., 2014)
11	3-O-ß-D-glucopyranosyl-3-epiruscogenin	C_41_H_60_O_12_	roots	(Park et al., 2014)
12	3-O-ß-D-glucopyranosyl-3-epineoruscogenin	C_41_H_58_O_12_	roots	(Park et al., 2014)
13	dancosterol	C_35_H_60_O_6_	tubers	(Han et al., 2001)
14	2,7-dihydroxy-4-methoxyphenanthrene-2-O-glucoside	C_21_H_22_O_8_	tubers	(Yamaki et al., 1993b)
15	2,7-dihydroxy-4-methoxyphenanthrene-2,7-O-diglucoside	C_27_H_32_O_13_	tubers	(Yamaki et al., 1993b)
16	3,7-dihydroxy-2,4-dimethoxyphenanthrene-3-O-glucoside	C_22_H_24_O_9_	tubers	(Yamaki et al., 1993b)
17	gastrodin	C_13_H_18_O_7_	fibrous roots	(Yu et al., 2016)
18	2,7-dihydroxy-l-(4’-hydroxybenzyl)-9,10-dihydrophenan-threne-4’-O-glucoside	C_28_H_30_O_9_	tubers	(Yamaki et al., 1993b)
19	3’-hydroxy-5-methoxybibenzyl-3-O-ß-D-glucopyranoside	C_21_H_26_O_8_	tubers	(Han et al., 2002b)
***Bibenzyls***				
20	blestritin A	C_37_H_36_O_6_	tubers	(Feng et al., 2008)
21	blestritin B	C_30_H_30_O_6_	tubers	(Feng et al., 2008)
22	blestritin C	C_36_H_34_O_6_	tubers	(Feng et al., 2008)
23	bulbocodin	C_36_H_34_O_6_	tubers	(Feng et al., 2008)
24	bulbocodin C	C_29_H_28_O_5_	tubers	(Ma et al., 2017)
25	bulbocodin D	C_29_H_28_O_5_	tubers	(Feng et al., 2008)
26	bulbocol	C_23_H_24_O_4_	tubers	(Feng et al., 2008)
27	gymconopin D	C_23_H_24_O_4_	tubers	(Feng et al., 2008)
28	shancigusin B	C_28_H_26_O_5_	tubers	(Ma et al., 2017)
29	shanciguol	C_28_H_26_O_5_	tubers	(Ma et al., 2017)
30	arundinan	C_22_H_22_O_3_	tubers	(Ma et al., 2017)
31	arundin	C_29_H_28_O_4_	tubers	(Ma et al., 2017)
32	batatasin III	C_15_H_16_O_3_	tubers	(Woo et al., 2014)
33	gigantol	C_16_H_18_O_4_	tubers	(Woo et al., 2014)
34	3,4’-dihydroxy-5,3’,5’-trimethoxybibenzyl	C_17_H_20_O_5_	tubers	(Woo et al., 2014)
35	3,3’-dihydroxy-5,4’-dimethoxybibenzyl	C_16_H_18_O_4_	tubers	(Feng et al., 2008)
36	3’-O-methylbatatasin III	C_15_H_18_O_3_	tubers	(Feng et al., 2008)
37	3,3’-dihydroxy-4-(p-hydroxybenzyl)-5-methoxybibenzyl	C_22_H_22_O_4_	tubers	(Bai et al., 1993)
38	3,3’-dihydroxy-2-(p-hydroxybenzyl)-5-methoxybibenzyl	C_22_H_22_O_4_	tubers	(Bai et al., 1993)
39	3’,5-dihydroxy-2-(p-hydroxybenzyl)-3-methoxybibenzyl	C_22_H_22_O_4_	tubers	(Bai et al., 1993)
40	2’,6’-bis(p-hydroxybenzyl)-5-methoxybibenzyl-3,3’-diol	C_33_H_36_O_5_	tubers	(Takagi et al., 1983)
41	2,6-bis(p-hydroxybenzyl)-5,3’-dimethoxybibenzyl-3-ol	C_30_H_30_O_5_	tubers	(Takagi et al., 1983)
42	3,3’-dihydroxy-5-methoxy-2,5’,6-tris(p -hydroxybenzyl) bibenzyl	C_46_H_44_O_11_	tubers	(Takagi et al., 1983)
43	3,3’,5-trimethoxybibenzyl	C_17_H_20_O_3_	tubers	(Yamaki et al., 1991)
44	3,5-dimethoxybibenzyl	C_16_H_18_O_2_	tubers	(Yamaki et al., 1991)
45	5-hydroxy-4-(p-hydroxybenzyl)-3’,3-dimethoxybibenzyl	C_23_H_24_O_4_	tubers	(Han et al., 2002a)
46	3,3’-dihydroxy-5-methoxybibenzyl	C_15_H_16_O_3_	tubers	(Han et al., 2002c)
47	5-hydroxy-2-(p-hydroxybenzyl)-3-methoxybibenzyl	C_22_H_22_O_3_	tubers	(Ma et al., 2017)
***Phenanthrenes***				
48	4-methoxyphenanthrene-2,7-diol	C_15_H_12_O_3_	tubers	(Feng et al., 2008)
49	3,4-dimethoxyphenanthrene-2,7-diol	C_16_H_14_O_4_	tubers	(Feng et al., 2008)
50	2,4-dimethoxyphenanthrene-3,7-diol	C_16_H_14_O_4_	tubers	(Feng et al., 2008)
51	3,5-dimethoxyphenanthrene-2,7-diol	C_16_H_14_O_4_	tubers	(Xiao et al., 2016)
52	1,5-dimethoxyphenanthrene-2,7-diol	C_16_H_14_O_4_	tubers	(Xiao et al., 2016)
53	2,4-dimethoxyphenanthrene-7-ol	C_15_H_14_O_3_	tubers	(Xiao et al., 2016)
54	2,4,7-trimethoxyphenanthrene	C_17_H_16_O_3_	tubers	(Yamaki et al., 1991)
55	2,3,4,7-tetramethoxyphenanthrene	C_18_H_18_O_4_	tubers	(Yamaki et al., 1991)
56	1,8-bis(p-hydroxybenzyl)-4-methoxyphenanthrene-2,7-diol	C_29_H_24_O_5_	tubers	(Bai et al., 1991)
57	1-(p-hydroxybenzyl)-4,8-dimethoxyphenanthrene-2,7-diol	C_23_H_20_O_5_	tubers	(Morita et al., 2005)
58	1-(p-hydroxybenzyl)-4-methoxyphenanthrene-2,7-diol	C_22_H_18_O_4_	tubers	(Yamaki et al., 1990)
59	2-hydroxy-4,7-dimethoxyphenanthrene	C_16_H_14_O_3_	fibrous roots	(Yu et al., 2016)
60	3,7-dihydroxy-2,4,8-trimethoxyphenanthrene	C_17_H_16_O_5_	tubers	(Woo et al., 2014)
61	2,7-dihydroxy-3,4-dimethoxyphenanthrene	C_16_H_14_O_4_	tubers	(Han et al., 2002c)
62	1-(p-hydroxybenzyl)-4,7-dimethoxyphenanthrene-2-ol	C_23_H_20_O_4_	tubers	(Xiao et al., 2017)
63	1-(p-hydroxybenzyl)-4,7-dimethoxyphenanthrene-2,8-diol	C_23_H_20_O_5_	tubers	(Xiao et al., 2017)
64	1-(p-hydroxybenzyl)-4,7-dimethoxyphenanthrene-2,6-diol	C_23_H_20_O_5_	tubers	(Xiao et al., 2017)
65	bleformin B	C_23_H_20_O_5_	tubers	(Ma et al., 2017)
66	blespirol	C_25_H_18_O_5_	tubers	(Yamaki et al., 1993b)
***Quinones***				
67	1,8-dihydroxy-3-methoxy-6-methylanthracene-9,10-dione	C_16_H_12_O_5_	tubers	(Wang et al., 2001)
68	2-methylanthraquinone	C_15_H_10_O_2_	tubers	(Sun et al., 2016c)
69	4,7-dimethoxyphenanthrene-1,2-dione	C_16_H_13_O_4_	tubers	(Xiao et al., 2016)
70	7-hydroxy-2-methoxyphenanthrene-3,4-dione	C_15_H_13_O_4_	tubers	(Sun et al., 2016a)
71	3’,7’,7-trihydroxy-2,2’,4’-trimethoxy-[1,8’-biphenanthrene]-3,4-dione	C_31_H_23_O_8_	tubers	(Sun et al., 2016a)
***Biphenanthrenes***				
72	blestrin A	C_30_H_26_O_6_	tubers	(Bai et al., 1990)
73	blestrin B	C_30_H_26_O_6_	tubers	(Bai et al., 1990)
74	blestrin C	C_30_H_24_O_6_	tubers	(Yamaki et al., 1992)
75	blestrin D	C_30_H_24_O_6_	tubers	(Yamaki et al., 1992)
76	blestriarene A	C_30_H_26_O_6_	tubers	(Yamaki et al., 1989)
77	blestriarene B	C_30_H_24_O_6_	tubers	(Yamaki et al., 1989)
78	blestriarene C	C_30_H_22_O_6_	tubers	(Yamaki et al., 1989)
79	blestrianol A	C_30_H_26_O_6_	tubers	(Bai et al., 1991)
80	blestrianol B	C_37_H_32_O_7_	tubers	(Bai et al., 1991)
81	blestrianol C	C_37_H_30_O_7_	tubers	(Bai et al., 1991)
82	4,7,3’5’-tetramethoxy-9’,10’-dihydro-[1,2’-biphenanthrene]-2,7’-diol	C_32_H_27_O_6_	fibrous roots	(Qian et al., 2015)
83	4,7,7’-trimethoxy-9’,10’-dihydro-[1,3’-biphenanthrene]-2,2’,5’-triol	C_31_H_25_O_6_	fibrous roots	(Qian et al., 2015)
84	4,7,4’-trimethoxy-9’,10’-dihydro-[1,1’-biphenanthrene]-2,2’,7’-triol	C_31_H_25_O_6_	fibrous roots	(Qian et al., 2015)
85	4,7,3’,5’-tetramethoxy-9’,10’-dihydro-[1,1’-biphenanthrene]-2,2’,7’-triol	C_32_H_27_O_7_	fibrous roots	(Qian et al., 2015)
86	4,8,4’,8’-tetramethoxy-[1,1’-biphenanthrene]-2,7,2’,7’-tetrol	C_32_H_26_O_8_	fibrous roots	(Qian et al., 2015)
87	bleformin D	C_37_H_32_O_7_	tubers	(Ma et al., 2017)
88	4,4’-dimethoxy-9,10-dihydro-[6,1’-biphenanthrene]-2,7,2’,7’-tetraol	C_30_H_24_O_6_	tubers	(Ma et al., 2017)
89	gymconopin C	C_30_H_26_O_6_	tubers	(Ma et al., 2017)
***Dihydrophenanthrenes***				
90	4,7-dihydroxy-2-methoxy-9,10-dihydrophenanthrene	C_15_H_14_O_3_	tubers	(Yamaki et al., 1990)
91	2,7-dihydroxy-3-(p-hydroxybenzyl)-4-methoxy-9,10-dihydrophenanthrene	C_22_H_20_O_4_	tubers	(Yamaki et al., 1990)
92	4,7-dihydroxy-1-(p-hydroxybenzyl)-2-methoxy-9,10-dihydrophenanthrene	C_22_H_20_O_4_	tubers	(Yamaki et al., 1990)
93	2,7-dihydroxy-1,6-bis(p-hydroxybenzyl)-4-methoxy-9,10-dihydrophenanthrene	C_29_H_26_O_5_	tubers	(Yamaki et al., 1990)
94	2,7-dihydroxy-l,3-bis(p-hydroxybenzyl)-4-methoxy-9,10-dihydrophenanthrene	C_29_H_26_O_5_	tubers	(Bai et al., 1993)
95	2,7-dihydroxy-l-(p-hydroxybenzyl)-4-methoxy-9,10-dihydrophenanthrene	C_22_H_20_O_4_	tubers	(Bai et al., 1993)
96	2,4,7-trimethoxy-9,10-dihydrophenanthrene	C_17_H_18_O_3_	tubers	(Yamaki et al., 1991)
97	2,7-dihydroxy-4-methoxy-9,10-dihydrophenanthrene	C_15_H_14_O_3_	tubers	(Han et al., 2002c)
98	4,5-dihydroxy-2-methoxy-9,10-dihydrophenanthrene	C_15_H_14_O_3_	fibrous roots	(Yu et al., 2016)
99	2,8-dihydroxy-4,7-dimethoxy-9,10-dihydrophenanthrene	C_15_H_14_O_3_	tubers	(Woo et al., 2014)
100	2,8-dihydroxy-1-(p-hydroxybenzyl)-4,7-dimethoxy-9,10-dihydrophenanthrene	C_23_H_22_O_5_	tubers	(Woo et al., 2014)
101	pleionesin C	C_27_H_26_O_7_	rhizomes	(Li et al., 2008a)
102	(2,3-trans)-2-(4-hydroxy-3-methoxyphenyl)-3-hydroxymethyl-10-methoxy-2,3,4,5-tetrahydro-phenanthro[2,1-b]furan-7-ol	C_25_H_24_O_6_	rhizomes	(Li et al., 2008a)
103	bleochranol A	C_40_H_38_O_8_	rhizomes	(Li et al., 2008a)
104	bleochranol B	C_25_H_24_O_6_	rhizomes	(Li et al., 2008a)
105	bleochranol C	C_33_H_32_O_8_	rhizomes	(Li et al., 2008a)
106	bleochranol D	C_34_H_32_O_8_	rhizomes	(Li et al., 2008a)
107	(2,3-trans)-3-[2-hydroxy-6-(3-hydro-xyphenethyl)-4-methoxybenzyl]-2-(4-hydroxy-3-methoxyphenyl)-10-methoxy-2,3,4,5-tetrahydrophenanthro[2,1-b]furan-7-ol	C_40_H_38_O_8_	rhizomes	(Li et al., 2008a)
108	shanciol	C_25_H_24_O_6_	tubers	(Ma et al., 2017)
109	bletlos A	C_28_H_28_O_8_	tubers	(Yamaki et al., 1993a)
110	bletlos B	C_27_H_26_O_7_	tubers	(Yamaki et al., 1993a)
111	bletlos C	C_27_H_26_O	tubers	(Yamaki et al., 1993a)
112	blestriaren A	C_30_H_26_O_6_	rhizomes	(Li et al., 2008a)
***Anthocyanins***				
113	Bletilla anthocyanin 1	C_75_H_81_O_40_	flowers	(Saito et al., 1995)
114	Bletilla anthocyanin 2	C_72_H_79_O_37_	flowers	(Saito et al., 1995)
115	Bletilla anthocyanin 3	C_75_H_81_O_43_	flowers	(Saito et al., 1995)
116	Bletilla anthocyanin 4	C_72_H_79_O_40_	flowers	(Saito et al., 1995)
117	3-O-(ß-glucopyranoside)-7-O-[6-O-(4-O-(6-O-(4-O-(ß-glucopyranosyl)-trans-caffeoyl)-ß-glucopyranosyl)-trans-caffeoyl)-ß-glucopyranoside]	C_57_H_63_O_32_	flowers	(Tatsuzawa et al., 2010)
***Steroids***				
118	ß-sitosterol	C_29_H_50_O	tubers	(Han et al., 2001)
119	ß-sitosterol palmitate	C_45_H_80_O_2_	tubers	(Yamaki et al., 1997)
120	stigmasterol	C_29_H_48_O	tubers	(Sun et al., 2016c)
121	stigamasterol palmitate	C_45_H_78_O_2_	tubers	(Yamaki et al., 1997)
122	3-epiruscogenin	C_27_H_42_O_4_	roots	(Park et al., 2014)
123	3-epineoruscogenin	C_27_H_40_O_4_	roots	(Park et al., 2014)
124	(20S,22R)-1ß,2ß,3ß,4ß,5ß,7α-hexahydroxyspirost-25(27)-en-6-one	C_27_H_35_O_9_	roots	(Park et al., 2014)
125	(1α,3α)-1-O-[(ß-D-xylopyranosyl-(1→2)-α-L-rhamnopyranosyl)]-3-O-D-glucopyranosyl-5α-spirostan	C_44_H_71_O_17_	roots	(Wang and Meng 2015)
126	(1α,3α)-1-O-[(ß-D-xylopyranosyl-(1→2)-α-L-rhamnopyranosyl)oxy]-3-O-D-glucopyranosyl-25(27)-ene-5α-spirostan	C_44_H_69_O_17_	roots	(Wang and Meng 2015)
127	(1α,3α)-1-O-[(ß-D-xylopyranosyl-(1→2)-α-L-rhamnopyranosyl)oxy]-epiruscogenin	C_38_H_59_O_12_	roots	(Wang and Meng 2015)
128	(1α,3α)-1-O-[(ß-D-xylopyranosyl-(1→2)-α-L-rhamnopyranosyl)oxy]-epineoruscogenin	C_38_H_57_O_12_	roots	(Wang and Meng 2015)
***Triterpenoids***				
129	cyclomargenol	C_32_H_54_O	tubers	(Yamaki et al., 1997)
130	cyclomargenone	C_32_H_53_O	tubers	(Yamaki et al., 1997)
131	cycloneolitsol	C_32_H_54_O	tubers	(Yamaki et al., 1997)
132	cyclobalanone	C_32_H_53_O	tubers	(Yamaki et al., 1997)
133	24-methylenecycloartanol palmitate	C_47_H_81_O_2_	tubers	(Yamaki et al., 1997)
134	cyclolaudenol	C_31_H_51_O	tubers	(Yang et al., 2014)
135	cyclolaudenone	C_31_H_50_O	tubers	(Yang et al., 2014)
136	3ß-hydroxyoleane-12-en-28-oic acid 3-O-α-L-rhamnopyranosyl-(1→2)-ß-D-glucopyranoside	C_41_H_55_O_12_	tubers	(Sun et al., 2016b)
***Phenolic acids***				
137	p-hydroxybenzoic acid	C_7_H_6_O_3_	tubers	(Takagi et al., 1983)
138	protocatechuic acid	C_7_H_6_O_4_	tubers	(Takagi et al., 1983)
139	cinnamic acid	C_9_H_8_O_2_	tubers	(Takagi et al., 1983)
140	caffeic acid	C_9_H_8_O_4_	tubers	(Han et al., 2001)
141	2-hydroxysuccinic acid	C_4_H_5_O_5_	tubers	(Sun et al., 2016b)
142	palmitic acid	C_16_H_32_O_2_	tubers	(Sun et al., 2016b)
143	syringaresinol	C_22_H_26_O_8_	tubers	(Han et al., 2001)
144	pinoresinol	C_20_H_22_O_6_	tubers	(Bae et al., 2016)
145	3’’-methoxynyasol	C_18_H_17_O_3_	tubers	(Bae et al., 2016)
146	p-hydroxybenzaldehyde	C_7_H_6_O_2_	tubers	(Takagi et al., 1983)
147	ferulic acid	C_10_H_10_O_4_	tubers	(Yu et al., 2011)
148	3-hydroxycinnamic acid	C_9_H_8_O_3_	tubers	(Yu et al., 2011)
***Others***				
149	4-hydroxybenzylamine	C_7_H_9_NO	tubers	(Sun et al., 2016b)
150	4,4’-dihydroxydiphenylmethane	C_13_H_12_O_2_	tubers	(Yan et al., 2014)
151	4,4’-dihydroxybenzyl sulfide	C_14_H_14_SO_2_	tubers	(Yan et al., 2014)
152	5-(hydroxymethyl)-2-furaldehyde	C_6_H_6_O_3_	tubers	(Sun et al., 2016c)
153	striatolide	C_18_H_30_O_3_	tubers	(Yan et al., 2014)
154	schizandrin	C_24_H_32_O_7_	tubers	(Han et al., 2002a)
155	brugnanin	C_56_H_90_O_8_	tubers	(Sun et al., 2016c)
156	bletillanol A	C_18_H_21_O_5_	tubers	(Bae et al., 2016)
157	bletillanol B	C_18_H_20_O_5_	tubers	(Bae et al., 2016)
158	tupichinol A	C_17_H_18_O_4_	tubers	(Bae et al., 2016)

### Glycosides

Glycosides are an important class of compounds in *B. striata* (**Figure 1**), of which polysaccharides are relatively abundant in its dried tubers. *B. striata* contains various natural monosaccharide compounds (**1–19**). *Bletilla striata* polysaccharide (BSP) is a high-molecular-weight, viscous polysaccharide, whose chemical composition is glucomannan (which comprises *D-*glucose and *D-*mannose) (Liu et al., 2013).

### Bibenzyls

Although the skeleton of bibenzyl compounds is simple, the bridge chain substituents attached to the aromatic ring are diverse. This scenario leads to various structural types and, thus, to various biologic activities (Dai and Sun, 2008). The structure of bibenzyl compounds is unique, and this is of considerable practical importance for finding benzyl compounds with potential medicinal value. *B. striata* is rich in benzyl compounds (**20–47**), containing 28 types, as shown in **Figure 2**.

### Phenanthrenes

Several studies have reported that phenanthrenes are present in *B. striata.* The substituents on the aromatic rings are mainly methoxy, hydroxyl, and *p*-hydroxybenzyl groups, as shown in **Figure 3**.

### Quinones

Benzoquinones, naphthoquinones, anthraquinones, and phenanthraquinones are commonly found in plants used for TCM. Five types of quinones have been isolated from *B. striata*, including anthraquinones (**67–68**) and phenanthraquinones (**69–71**), whose structures are shown in **Figure 4**.

### Biphenanthrenes

Due to their axial asymmetry and asymmetric induction, biphenanthrene compounds exhibit important characteristic absorption in the infrared region at 1620–1480 cm^−1^ and 900–650 cm^−1^ (Tang et al., 2014). The biphenanthrene compounds in *B. striata* are composed of two simple phenanthrenes or dihydrophides, as shown in **Figure 5**.

### Dihydrophenanthrenes

Dihydrophenanthrenes, phenanthrenes, and benzyls are stilbenoids with an identical 1,2-distyrene skeleton. The substituents on their aromatic rings are mainly hydroxyl, methoxy, and *p*-hydroxybenzyl groups (Lin et al., 2005). In the ultraviolet spectrum, dihydrophenanthrene compounds, in general, have an α band at approximately 310 nm, a β band at approximately 250 nm, and a ρ band at approximately 280 nm. The skeleton of dihydrophenanthrenes can be synthesized in the phenylpropane metabolic pathway using phenolic compounds (Gu et al., 2013). The structures of dihydrophenanthrenes are shown in **Figure 6**.

### Anthocyanins

Anthocyanins are the main pigments that give flowers their color. The flowers of *B. striata* are not commonly used in TCM, and therefore, few studies have been conducted on these flowers. Only five compounds (**113–117**) have been isolated from the flowers of *B. striata*. However, recent studies have shown that the anthocyanins in plants may affect human health (Stintzing and Carle, 2004), and have provided new material to study the medicinal uses of *B. striata*. The structures of anthocyanins are shown in **Figure 7**.

### Steroids

All steroids are biosynthesized from mevalonic acid (Debieu et al., 1992). The steroids present in *B. striata* are complex, with diverse structures and many derivatives. Eleven steroids and their derivatives (**118–128**) have been isolated from *B. striata*, and their structures are shown in **Figure 8**.

### Triterpenoids

Triterpenoids are widespread in plants. They are mostly tetracyclic and pentacyclic, but a few are monocyclic, bicyclic, and tricyclic (Liou and Wu, 2002). The triterpenoids isolated from *B. striata* have mainly tetracyclic triterpene structures (**129–135**), but also include a pentacyclic triterpene branched diglycoside compound (**136**). Their structures are shown in **Figure 9**.

### Phenolic Acids

Phenolic acids are organic acids containing phenol rings, hydroxybenzoic acids, and hydroxycinnamic acids. They are important secondary metabolites in plants because of the protection they offer against insects, viruses, and bacteria (Heleno et al., 2015). Consumption of phenolic acid-rich food could assist with accelerating the elimination of oxygen free radicals in the body, which would protect cells (Bae et al., 2016). Twelve phenolic acids (**137–148**) have been obtained from *B. striata*, and their structures are shown in **Figure 10**.

### Other Compounds

In addition to the compounds stated above, other compounds from *B. striata* with different structures (**149–158**) have been reported. Their structures are shown in **Figure 11**.

## Pharmacologic Activities

The basic research of TCM focuses mainly on chemical composition and pharmacology, of which the latter is the most important. Pharmacologic studies on *B. striata* have mainly focused on pharmacokinetics. The latter advances our understanding of the mechanism of action of *B. striata* components. In recent years, pharmacologic studies on *B. striata* have focused mainly on its hemostatic, wound-healing, anti-oxidative, anti-cancer, antiviral, and antibacterial activities.

### Hemostasis

Hemostasis is a process that causes bleeding to stop (i.e., blood is retained within a damaged blood vessel). The hemostatic effect is one of the main pharmacologic effects of *B. striata.* Bensky and colleagues showed that when the dried powder of *B. striata* tubers was mixed with water, it could achieve a good hemostatic effect after it was spread onto a wound (Venkatraja et al., 2012). Interestingly, recent studies have shown that the water-soluble portion of *B. striata* has an active role in hemostasis, and its function is believed to be related to adenosine diphosphate, which promotes and accelerates platelet aggregation (Lu et al., 2005; Gachet, 2006). Hung and Wu found that BSP in this water-soluble component played a key role in hemostatic activity (Hung and Wu, 2016). Indeed, there has recently been intensive research on the procoagulant function of BSP.

Blood coagulation is a process in which a series of coagulation factors is successively activated by enzymatic action to produce thrombin and fibrinogen clots. Animal experiments have shown that BSP can participate in multiple hemostasis processes, such as platelet adherence to the subendothelial matrix to block vessels (primary hemostasis), and the formation of fibrin clots (secondary hemostasis) by activation of various coagulation factors and promotion of thromboxane-A2 synthesis (Dong et al., 2014). Because of its obvious and non-toxic hemostatic effect, BSP has been developed as a new type of hemostatic agent that can be used as a drug delivery vehicle and wound dressing (Zhang et al., 2017a). BSP can be combined with other materials to develop new biomedical materials, such as hemostatic materials for surgical treatments (Wang et al., 2017; Chen et al., 2019).

In addition to BSP, the steroids (**123, 125–128**) in *B. striata* exhibit obvious hemostatic activity and can significantly reduce clotting time (Yamaki et al., 1990). The hemostatic effect of this type of composition may be related to platelets, blood clotting, and fibrinolysis (Zhao et al., 2016).

### Wound Healing

Wound healing has three main phases: inflammation, proliferation of granulation tissue, and repair (Kasuya and Tokura, 2014). Antioxidants in plants, such as polysaccharides and phenolic compounds, have important roles in these three phases (Süntar et al., 2012).

In the inflammation phase, BSP promotes the expression of cytokines such as tumor necrosis factor (TNF)-α, interleukin (IL)-1β, and interferon (IFN)-γ (Diao et al., 2008). As downstream effector molecules of the toll-like receptor-4/lipopolysaccharide (TLR4/LPS) signaling pathway, TNF-α and IL-1β are mediators of the inflammatory response. They have similar effects and can activate various inflammatory cells (Cunha et al., 2007). Also, IFN-γ enhances the expression of major histocompatibility complex class-II molecules on macrophage surfaces to improve their ability to present antigens (Baldridge et al., 2010). Additionally, it was found that a BSP solution (11 μM) increased the nitric oxide (NO) concentration in a wound, which would promote the chemotaxis of neutrophils, monocytes, and macrophages, thereby providing adequate conditions for wound repair (Diao et al., 2008). NO can also regulate the diameter of and flow within blood vessels so that the wound obtains a more generous blood supply, which helps to repair the wound and advance the growth of new blood vessels. Studies have indicated that BSP can promote the cleanliness of necrotic tissue and provide conditions for subsequent tissue regeneration in a wound (Huang et al., 2019).

In the phases based on the proliferation of granulation tissue and repair, BSP can control the expression of pro-inflammatory factors (e.g., TNF-α) at an appropriate level, reduce the inflammatory reaction in the wound, and prevent damage to remaining cells (Lou et al., 2010). In addition, BSP promotes an increase in the expression of the vascular endothelial growth factor (VEGF), as well as the synthesis and release of hydroxyproline. These actions promote the growth of epithelial cells, accelerate fibroblast proliferation, and further promote healing by wound contraction (Wang et al., 2006; Yu et al., 2011).

Apart from BSP, several phenolic acids (**137–138, 140, 146–148**) in *B. striata* are thought to promote wound healing (Song et al., 2017). Yang and colleagues reported that the esterification products of caffeic acid (**140**) and phenylethyl alcohol can inhibit the transforming growth factor-β1/Mothers against the decapentaplegic homolog 3 (TGF-β1/Smad3) signaling pathway (Yang et al., 2017). There is evidence that Smad3 can bind the DNA sequences of target genes at the transcriptional level, and, for pathologic skin conditions, assumes important roles in tissue repair and fibrosis (Ashcroft et al., 1999). Also, the degradation product of protocatechuic acid (**138**) can regulate high mobility group box (HMGB)1 expression by modulating the HMGB1/receptor for the advanced glycation end products (RAGE) pathway (Zhang et al., 2015). During wound healing, classically activated macrophages can utilize HMGB1 to attract vessel-associated stem cells (e.g., endothelial progenitor cells, vascular progenitor cells, smooth muscle progenitor cells), which contribute to skin healing and angiogenesis (Lolmede et al., 2009).

### Anti-Oxidation

Reactive oxygen species (ROS) have dual biologic effects. These are necessary to maintain cellular homeostasis during normal activities, but they also damage macromolecular matter. ROS over-accumulation results in cell death, can lead to multiple diseases, and accelerates the aging of the human body (Mittler, 2002). It is advantageous that various chemical constituents in plants can be used as natural antioxidants to remove free radicals in the body without the need for catalase, peroxidase, or superoxide dismutase (Grant and Loake, 2000).

Assays based on the scavenging of 2,2-diphenyl-1-picrylhydrazyl (DPPH) radicals have shown that the fibrous root parts and pseudobulb parts of *B. striata* exhibit strong free radical-scavenging activity. Indeed, the partial antioxidant capacity of chloroform subfractions from ethanol extracts of the fibrous roots of *B. striata* was the strongest (half-maximal inhibitory concentration (IC_50_) = 0.848 mg/L). Simultaneously, because the fibrous root parts contain more total phenols, they showed stronger reducing ability (reducing power RP_0.5AU_ = 83.68 mg/L) (Jiang et al., 2013).

BSP has been shown to be a natural antioxidant through various antioxidant test systems (DPPH, 2,2’-azino-bis, ferric-reducing antioxidant power). The antioxidative effects of BSP are mediated through the nicotinamide adenine dinucleotide phosphate oxidase 4 (NOX4)/p22^phox^ signaling pathway (Qu et al., 2016). Through this signaling pathway, BSP can inhibit expression of NOX4 and p22^phox^, thereby blocking angiotensin II-induced ROS generation (Yue et al., 2016). Furthermore, ferulic acid (**147**) in *B. striata* shows favorable free radical-scavenging ability, and its reducing ability is even stronger than that of vitamin C (Zhao et al., 2010). This antioxidant ability can protect cells from oxygen species-mediated DNA damage and radiation-induced free radicals (Srinivasan et al., 2006). The NOX4/ROS-mitogen-activated protein kinase pathway is the one through which ferulic acid (**147**) can decrease ethanol-induced ROS accumulation to improve anti-apoptotic responses (Li et al., 2017).

In some cases, antioxidants can worsen oxidative stress (Miller et al., 2005). If an antioxidant effect is observed, trace ROS are important cellular messengers of molecular signaling. Excessive clearance of ROS may lead to redox imbalance and induce cell-signaling disturbances, thereby triggering oxidative systems in the body.

### Anti-Cancer

The anti-cytotoxic activity of synthetic drugs can strengthen some of the medicinal effects of *B. striata*, especially efficacy against cancer-related diseases. Glycosides (**9–12**), bibenzyls (**30, 32–34, 38–41**), phenanthrenes (**57**), quinines (**70–71**), dihydrophenanthrenes (**90, 94, 98, 100–103, 105–107, 112**), steroids (**125–128**), and triterpenoids (**136**) from *B. striata* have been reported to show inhibitory activities against various tumor cell lines *in vitro*: HepG2, MCF-7, HT-29, A549, BGC-823, HL-60, MCF-7, SMMC-7721, W480, SK-OV-3, SK-MEL-2, and HCT-15. Their names and anti-cancer activities are listed in **Table 2**.

**Table 2:** Anti-cancer activities of *B. striata*.

Aspects	Compound No.	Tumor Cell	Anti-Tumor Activity Mechanism	References
Directly	100	HCT-15	Inhibit growth of tumor	(Woo et al., 2014)
	90, 98	HepG2	Arrest the cells at G2/M phase	(Wang et al., 2017)
	39, 40	K562	Cell cycle arrest	(Morita et al., 2005)
	70, 71	MCF-7, HT-29, A549	Induce apoptosis	(Sun et al., 2016a)
	136	A549	Arrest the cells at G0 phase	(Sun et al., 2016c)
	12, 125–128	A549, BGC-823, HepG2, HL-60, MCF-7, SMMC-7721,W480	Inhibit growth of tumor	(Wang and Meng, 2015)
	9–11	A549, SK-OV-3, SK-MEL-2, HCT-15	Inhibit growth of tumor	(Wang and Meng, 2015)
	30, 38, 40-41, 101-103, 105-107, 112	HL-60, SMMC-7721, A549, MCF-7, W480	Inhibit growth of tumor	(Li et al., 2008a)
Indirectly	40, 59, 94	K562	Reverse tumor multidrug resistance	Morita et al., 2005

Woo and colleagues revealed that compound **(100)** exhibited growth inhibition against a human colon cancer cell line (HCT-15), with an IC_50_ of 2.16 μM (Woo et al., 2014). Compounds (**90, 98**) isolated from *B. striata* tubers showed potent inhibition of proliferation of human hepatoma cells (HepG2), with an IC_50_ of 29.1 μM and 25.5 μM, respectively. In HepG2 cells, these two dihydrophenanthrenes induced apoptosis by downregulating the expression of cyclin B1 and blocking the cell cycle in the G2/M phase (Wang et al., 2017). Compounds **(40, 57, 94)** were shown to sensitize K562/breast cancer resistance protein (BCRP) cells to the metabolically active product camptothecin, SN-38, by inhibiting the function of BCRP cells, thereby demonstrating that they could alter multidrug resistance in cancer treatment (Morita et al., 2005). Compounds **(39, 40)** inhibited tubulin polymerization at an IC_50_ of 10 μM, and they limited cell growth by interfering with the mitosis of tumor cells (Morita et al., 2005). Compounds **(70–71)** exhibited significant cytotoxic effects against the MCF-7, HT-29, and A549 lines. After MCF-7 cells underwent treatment, the IC_50_ for compounds **(70–71)** was 18.49 μg/ml and 12.64 μg/ml, which are greater values than that for cisplatin. Interestingly, their anti-cancer activity is related to their pro-oxidative activity.

These compounds can block the G0/G1 phase *via* a ROS-mediated mechanism that ultimately leads to apoptosis (Sun et al., 2016a). Compound **(136)** has been shown to inhibit the proliferation of tumor cells in the same manner by blocking the G0/G1 phase (Sun et al., 2016c). The IC_50_ for compounds **(12, 125–128)** against A549, BGC-823, HepG2, HL-60, MCF-7, SMMC-7721, and W480 cells ranged from 11.3 μM to 32.2 μM, a range that is similar to that for doxorubicin (Wang and Meng, 2015). Compounds **(9–11)** showed significant cytotoxicity against A549, SK-OV-3, SK-MEL-2, and HCT-15 cells, with IC_50_ ranging from 3.98 μM to 12.10 μM (Wang and Meng, 2015). Compounds **(30, 38, 40–41, 101–103, 105–107, 112)** showed significant cytotoxicity against HL-60, SMMC-7721, A549, MCF-7, and W480 cells with IC_50_ ranging from 0.24 μM to 38.56 μM (Li et al., 2008a). Among them, compound **(103)** showed stronger cytotoxicity than cisplatin against the cell lines mentioned above.

In addition to the monomeric compounds stated above, BSP is also considered to have anti-cancer activity (Zhang et al., 2019b). Qian and colleagues showed that, compared with transcatheter arterial chemoembolization (TACE) alone, better results were obtained when the hepatocellular carcinoma in ACI rats was treated with a combination of TACE and arterial administration of BSP (Qian et al., 2003). BSP also inhibited the proliferation of HepG2 cells in a weak manner, and the division of HepG2 cells was not inhibited at <1.5 mg/ml. BSP can also induce apoptosis through caspase-3 expression (Liu et al., 2018). Using BSP as a drug carrier or mixing chemotherapeutic drugs with BSP is another anti-cancer strategy. In this manner, the drug concentration in the target organ can be maintained at a particular level. Based on this idea, copolymer micelles have been used in cancer chemotherapy (Wang et al., 2019). A macromolecular substance consisting of stearic acid-modified BSP as a carrier of docetaxel was created, and had a more pronounced effect on inhibiting the growth of HepG2 and HeLa cancer cells compared with that of docetaxel injection alone (Guan et al., 2017).

### Antiviral

The efficacy of several first-line antiviral agents appears to be diminishing (Ruiz and Russell, 2012). The antiviral effect of some traditional Chinese herbs (e.g., *Isatis tinctoria*) has been demonstrated by experimental and clinical research (Li and Peng, 2013). The active pharmaceutical ingredient in these plants can be used as the lead compound for further structural optimization to develop new antiviral drugs that can strengthen the immune system to fight and eliminate viral infections.

*B. striata* can exert antiviral activity by (i) interfering with the surface proteins of viruses to reduce cell infection and inhibit virus invasion into tissue; (ii) interfering with the RNA replication of the invading viruses to inhibit viral proliferation in the body; and (iii) preventing the virus from being released from host cells, thereby reducing viral spread (Shi et al., 2017; Zhang et al., 2017b). The water and ethanol extracts of *B. striata* avert invasion by the influenza virus by interfering with the hemagglutinin receptor on Madin-Darby canine kidney (MDCK) cells, and viral inhibition increases with the extract concentration, with an IC_50_ of 18.3 mg/ml and 235.7 μg/ml, respectively, being recorded (Zhang et al., 2017b). Various compounds (**53**, **84–86**, **88**, **90**, **97–98**) in *B. striata* also show different levels of antiviral activity. Most of them have been shown to have significant antiviral activity against the H3N2 virus in an embryonated hen-egg model, with inhibition ranging from 17.2% to 79.3% (Shi et al., 2017).

*B. striata* can also assist the immune system of the human body to indirectly exert antiviral activity. Peng and colleagues investigated the immunomodulatory activity of BSPF2, which is a new polysaccharide identified from *B. striata*. BSPF2 significantly induced spleen cell proliferation in a dose-dependent manner (Peng et al., 2014). The spleen can produce lymphocytes, macrophages, and various cytokines, and has an important role in the immune system. Animal experiments have demonstrated that *B. striata* can promote T-cell and B-cell immunity in immunocompromised mice (Qiu et al., 2011).

### Antibacterial

The use of phytochemicals could solve the problems of drug-resistant strains and antibiotic shortages. Phytochemicals are safe, low-toxicity agents with important bacteriostatic functions (Barbieri et al., 2017).

Testing of the antibacterial activity of *B. striata* has revealed that bibenzyls (**40, 42**) and biphenanthrenes (**76–78, 83–86**) are active against Gram-positive and Gram-negative strains. Their names and antibacterial activities are listed in **Table 3**. Compound (**84**) exhibited potential activity against *Staphylococcus aureus* American Type Culture Collection (ATCC) 29213, methicillin-resistant *S. aureus* (MRSA) ATCC 43300, and *Enterococcus faecalis* ATCC 29212 with a minimum inhibitory concentration (MIC) of 2–8 µg/ml. Scanning electron microscopy showed that compound (**84**) killed bacterial cells by damaging their cytoplasmic membranes (Chen et al., 2018). Compounds of **78 and 83–86,** with a MIC of 2 μg/ml and 64 μg/ml, were active against six Gram-positive bacteria: *S. aureus* ATCC 25923, 29213, 43300, *Staphylococcus epidermidis* Center for Medical Culture Collections (CMCC) 26069, *E. faecalis* ATCC 29212, and *Bacillus subtilis* China General Microbiological Culture Collection Center (CGMCC) 1.1470 (Qian et al., 2015). Blestriarene A (**76**), blestriarene B (**77**), and blestriarene C (**78**) showed activity against *S. aureus* ATCC 25923 at MIC of 12.5–50 mg/ml, against *S. epidermidis* ATCC 26069 at MIC of 25–50 mg/ml, and against *B. subtilis* ATCC 6633 at MIC of 25 mg/ml (Yang et al., 2012). Compound (**40**) exhibited *in vitro* activity against *Bacillus cereus* ATCC 11778 and *S. aureus* ATCC 25923, with a MIC of 6.25 μg/ml, and the MIC for compound (**42**) against *S. aureus* ATCC 25923 was 3.12 μg/ml (Takagi et al., 1983). Furthermore, the phenanthrene fraction from the ethanol extract of *B. striata* has been regarded as a significantly active agent against Gram-positive bacteria, including clinical isolates of MRSA and *S. aureus* (*S. aureus* ATCC 25923, 29213, 43300) (Guo et al., 2016).

**Table 3:** Antibacterial activities of *B. striata*.

Compound No.	Bacteria	MCI	References
40	*C. albicans* ATCC 10257	> 100 µg/ml	(Takagi et al., 1983)
	*B.cereus* ATCC 11778	6.25 µg/ml	
	*S. aureus* ATCC 25923	6.25 µg/ml	
42	*C. albicans* ATCC 10257	> 100 µg/ml	(Takagi et al., 1983)
	*S. aureus* ATCC 25923	3.12 µg/ml	
76.77.78	*S. aureus* ATCC 25923	12.5–50 mg/ml	(Yang et al., 2012)
	*S. epidermidis* ATCC 26069	25–50 mg/ml	
	*B. subtilis* ATCC 6633	25 mg/ml	
78	*S. aureus* ATCC 25923	32 µg/ml	(Qian et al., 2015)
	*S. aureus* ATCC 29213	16 µg/ml	
	*S. aureus* ATCC 43300	16 µg/ml	
	*S. epidermidis* CMCC 26069	16 µg/ml	
	*E. faecalis* ATCC 29212	16 µg/ml	
	*E. coli* ATCC 35218	>128 µg/ml	
	*P. vulgaris* CMCC 49027	>128 µg/ml	
83	*S. aureus* ATCC 25923	8 µg/ml	(Qian et al., 2015)
	*S. aureus* ATCC 29213	8 µg/ml	
	*S. aureus* ATCC 43300	8 µg/ml	
	*S. epidermidis* CMCC 26069	8 µg/ml	
	*E. faecalis* ATCC 29212	64 µg/ml	
	*E. coli* ATCC 35218	>128 µg/ml	
	*P. vulgaris* CMCC 49027	>128 µg/ml	
84	*S. aureus* ATCC 25923	4 µg/ml	(Qian et al., 2015)
	*S. aureus* ATCC 29213	2 µg/ml	
	*S. aureus* ATCC 43300	4 µg/ml	
	*S. epidermidis* CMCC 26069	4 µg/ml	
	*E. faecalis* ATCC 29212	4 µg/ml	
	*E. coli* ATCC 35218	>128 µg/ml	
	*P. vulgaris* CMCC 49027	>128 µg/ml	
85	*S. aureus* ATCC 25923	64 µg/ml	(Qian et al., 2015)
	*S. aureus* ATCC 29213	32 µg/ml	
	*S. aureus* ATCC 43300	32 µg/ml	
	*S. epidermidis* CMCC 26069	8 µg/ml	
	*E. faecalis* ATCC 29212	>128 µg/ml	
	*E. coli* ATCC 35218	>128 µg/ml	
	*P. vulgaris* CMCC 49027	>128 µg/ml	
86	*S. aureus* ATCC 25923	16 µg/ml	(Qian et al., 2015)
	*S. aureus* ATCC 29213	8 µg/ml	
	*S. aureus* ATCC 43300	16 µg/ml	
	*S. epidermidis* CMCC 26069	8 µg/ml	
	*E. faecalis* ATCC 29212	64 µg/ml	
	*E. coli* ATCC 35218	>128 µg/ml	
	*P. vulgaris* CMCC 49027	>128 µg/ml	

## Clinical Application

### TCM Formulations

Based on classical theories of TCM, Chinese medicinal formulations are prepared by pooling different types of medicinal herbs together (Li and Peng, 2013). During the development of TCM theory, many formulations have been recorded in pharmacopeias or by folklore based on clinical experiences. Considering the different causes and symptoms of diseases, *B. striata* is often used with other drugs to offset the toxicity of one drug or enhance the bioavailability of another drug, which is known as the “correspondence of prescription and syndrome” in TCM.

For example, *Bai Ji San* (*B. striata* liniment) is frequently used as an astringent hemostatic medicine. It is composed of *B. striata* (Bai Ji in Chinese), *Asarum sieboldii* (Xi Xin), *Saposhnikovia divaricata* (Fang Feng), and *Semen Platycladi* (Bai Zi Ren), as noted in *Tai Ping Sheng Hui Fang* (medical literature edited by the Song Dynasty government). *Qu Huo Wai Xiao Tang* (decoction for purging fire) can be used to treat skin scalding. It is composed of *Bai Ji*, *Sanguisorba officinalis* (Di Yu), *Cacumen Platycladi* (Bai Ye), stir-baked *Fructus Gardeniae* (Chao Zhi Zi), *Cynanchum otophyllum* (Qing Yang Sheng), *Angelica sinensis* (Dang Gui), and *Radix Glycyrrhizae* (Gan Cao), as noted in *Dong Tian Ao Zhi* (*Practical Surgical and Clinical Experiences*). Although the pharmacologic mechanism of these formulations is not clear, considerable clinical evidence suggests that *B. striata* has value in the treatment of various diseases.

### Embolizing Agent

TACE is a first-line treatment for most inoperable tumors (Tsurusaki and Murakami, 2015). An embolizing agent based on *B. striata* and TACE has been used in clinical applications. The *B. striata* embolizing agent can, in general, be classified into three types according to its formulation: liquid, compound, or microspheres.

The *B. striata* liquid embolizing agent is composed of BSP, cellulose diacetate (solute), and dimethyl sulfoxide (solvent). It has the characteristics of easy flow, good biocompatibility, and no fixed morphology. It can be used for the embolization of irregular tumor cavities and reduces damage to vascular walls (Sun et al., 2005).

The *B. striata* compound embolizing agent is a synthetic moiety that can be used as an anti-carcinogen and coagulant. In this compound, *B. striata* can have a pharmacologic role with other medicinal ingredients. It can inhibit the proliferation and spread of tumor cells, and reduce the risk of adverse reactions (Chen et al., 2006).

*B. striata* polysaccharide microspheres (BSPMs) could be promising transarterial chemoembolization carriers for cancer treatment. BSPMs show favorable drug-loading, swelling, suspension, drug-entrapment, and release characteristics *in vitro*, which are conducive to long-term targeted chemotherapy (Li et al., 2018b). Studies have shown that BSPMs can embolize the blood supply of hepatic arteries and the hepatic portal vein, and completely inhibit the growth of tumors and surrounding microvessels by stopping the binding of VEGF to its receptor (Zhao et al., 2004).

The *B. striata* embolizing agent has also performed well in TACE of hepatic cirrhosis with portal hypertension and secondary hypersplenism. In a study by Liu and coworkers, long-term follow-up of surgical patients revealed a mean survival duration of 61.5 ± 9.1 (median, 60; range, 1–157) months in the control group and 63.4 ± 9.9 (52, 0–161) months in the *B. striata* group. The spleen thickness of treated patients was reduced compared with that before TACE, and the counts of white blood cells, platelets, and red blood cells returned to within normal ranges (Liu et al., 2011). Therefore, TACE using *B. striata* as an embolizing agent is a safe and efficacious treatment for patients with hepatic cirrhosis with portal hypertension and secondary hypersplenism.

### Mucosa-Protective Agent

The imbalance between proteases and mucosal defense factors is an important cause of several digestive-system diseases (e.g., gastric ulcers, duodenal ulcers, and ulcerative colitis). In recent years, strengthening mucosal defenses has become a new strategy to treat peptic ulcers (Al-Jiboury and Kaunitz, 2012). *B. striata* can be used as a mucosa-protective agent alone or in combination with other Chinese herbal medicines (Zhang et al., 2019a). It can be administered by gastroscopic spraying, injection through a gastric tube, oral administration, or enemas (Zhou et al., 2019).

In the treatment of diseases of the upper gastrointestinal tract, *B. striata* forms a protective film on the damaged mucosal surface to protect it from erosion (by gastric acid) and digestion (by pepsin), thus enhancing the defensive function of the gastric mucosa (He et al., 2016). A preliminary clinical trial showed that *B. striata* combined with *Panax notoginseng*, omeprazole, and amoxicillin could reduce the inflammatory response of gastric ulcers, protect the ulcer surface, and promote the regeneration and repair of ulcerated tissue (Bai and Zhang, 2010). In the treatment of ulcerative colitis, *B. striata* can be added to an enema to act as a mucosa-protective agent. After *B. striata* powder enters the intestine, it can form a gel on the surface of the intestinal mucosa to protect it. It can also enhance adhesion of drugs to the intestinal wall, thereby increasing the drug concentration in the intestinal tract.

*B. striata* has a hemostatic function and can promote ulcer healing, which can aid regeneration of the intestinal mucosa and, thus, promote the healing of ulcerative colitis (Lan and Chang, 2014). BSP can alleviate the pathologic damage and symptoms of ulcerative colitis. It can inhibit the expression of TNF-α and nuclear factor-kappa B, upregulate the expression of IL-10, prevent the abnormal release of proinflammatory factors, promote repair of the intestinal mucosa, and inhibit inflammation (Ke and Zhao, 2011). BSP can also reduce the expression of Th2 cytokines in the colon by inhibiting macrophage activation, and relieve inflammation in the intestinal tract. Furthermore, enemas containing *B. striata* can reduce the risk of postoperative complications of patients with an artificial anus in the colon, reduce the possibility of inflammatory reactions in the surrounding skin, reduce erosion and hemorrhage in the artificial anal mucosa, and improve the quality of life for patients (Lan and Chang, 2014).

### Novel Biomaterials

Recently, novel “dissolving microneedles” have been created using BSP. Microneedles can carry drugs into the skin for drug delivery and release encapsulated drugs over time. Compared with conventional transdermal patches and subcutaneous injections, *B. striata* polysaccharide microneedles are a minimally invasive method of drug delivery that prevents skin retention of biohazardous sharp waste. The pharmacologic activity of BSP contributes to the recovery from the micro-trauma caused by microneedles, such as prevention of bacterial infection and reduction of inflammation (Hu et al., 2018).

Mixing *B. striata* and polyvinyl alcohol to form a dressing matrix can result in a novel, high-quality biologic dressing. This material makes full use of the pharmacologic activity of *B. striata* for hemostasis and wound healing, and has been shown to have good mechanical properties and biocompatibility. Hence, it can absorb oozing blood and tissue fluid, which accelerates the healing of burns, surgical wounds, and acute wounds (Lin et al., 2012).

A novel, readily stripped bilayer composite was designed as a wound-dressing material, and exhibited excellent biocompatibility and mechanical properties. This composite comprised an upper layer of soybean protein nonwoven fabric coated with a lower layer of genipin-crosslinked chitosan and *B. striata* herbal extract. The extract in the wound-dressing material was non-toxic, but also promoted the growth of L929 fibroblasts, which are beneficial for wound healing (Liu and Huang, 2010).

### Quality Control

The China Food and Drug Administration defines “geo-authentic” herbs as traditional Chinese crude drugs grown in unadulterated environments and subjected to natural conditions, or with specific cultivation techniques and processing methods (Brinckmann, 2013). Zheng’an County in Guizhou Province is accepted as the most optimal location to produce the crude tubers of *B. striata* in China. The *B. striata* produced here is called “Zheng’an Bai Ji”. However, the material basis and potential mechanisms for producing geo-authentic herbs are not completely clear. The *Chinese Pharmacopoeia* recommends identifying the geo-genuine properties of *B. striata* according to morphologic, microscopic, and thin-layer chromatography approaches, and by ensuring that the residual inorganic components after ashing are ≤15.0% (He et al., 2016).

The standards mentioned above are accepted in formularies and pharmacopoeias, but may not be sufficient to evaluate the quality of all *B. striata* tubers. The latter contain various medicinally active ingredients (e.g., glycosides, bibenzyls, phenanthrenes, biphenanthrenes, and dihydrophenanthrenes) which should also be considered in quality control. For example, assessment of amounts of dactylorhin A (**1**), gymnoside V (**5**), gymnoside IX (**6**), militarine (**8**), and bletilnoside A (**9**) by ultra-high-performance liquid chromatography using photo-diode array detection showed obvious variation in response to the different origins of *B. striata*. The mass fraction of each chemical marker was 0.341–1.110, 2.840–6.990, 5.790–34.400, 0.191–3.890, and 0.184–5.050 mg/g, respectively. Among them, a wild strain of *B. striata* produced in Zheng’an County contained relatively high levels of active ingredients at 1.110, 6.500, 31.400, 3.890, and 5.05 mg/g, respectively (Wang et al., 2014). Furthermore, Liu et al. used a parallel-line assay based on quantitative responses to evaluate the hemostatic potency of *B. striata* obtained from different habitats, which provided monitoring indicators for the clinical efficacy of *B. striata* (Liu et al., 2014). These preliminary results may provide evidence for the geographic specificity and quality control of geo-authentic herbs.

### Toxicology

Toxic side effects and adverse reactions from *B. striata* have rarely been reported. If *B. striata* is used with *Aconitum carmichaeli*, it enhances the content of hypaconitine in the decoction (Weng et al., 2004). However, hypaconitine can increase the expression of RyR_2_ (a regulatory protein related to the function of calcium channels) in cardiac muscle, which may result in an abnormal heart rate (Liu et al., 2016). Therefore, *B. striata* cannot be used with *A. carmichaeli* in TCM (He et al., 2016).

Tests to determine the acute toxicity of *B. striata* have shown that the mean mortality of mice is <20% if a single intragastric dose is increased to 80 g/kg body weight, and the median lethal dose of *B. striata* was not detected. Subsequent experiments using a maximum dose of *B. striata* that did not cause the death of experimental animals was determined to be 180 g/kg body weight (Zhang et al., 2013).

A series of toxicology experiments showed that BSP did not elicit allergic reactions, phototoxicity reactions and, most importantly, no obvious adverse reactions to human skin. These experiments included tests of acute oral toxicity (in mice), skin stimulation (rabbits), skin allergy (guinea pigs), skin phototoxicity (guinea pigs), and skin patches (humans) (Zhang et al., 2003). Yue and colleagues showed that the acute toxicity of BSP was very low. Mice were given BSP (4 g/kg body weight, i.g.) twice during an interval of 6 h. None of the mice died, and no significant changes were detected in their activity, food intake, fur, or weight (Yue et al., 2003).

## Conclusions and Perspectives

*B. striata* has been used as a medicinal herb in China for thousands of years. However, due to the high degree of personalization of the diagnosis and treatment of TCM, its clinical efficacy cannot be comprehensively evaluated by evidence-based medicine. In this review, we categorized research on *B. striata* based on its chemical constituents, pharmacologic activities, and clinical applications. We also tried to establish connections between the conclusions of many studies carried out on *B. striata*.

More than 150 compounds have been isolated from various parts of *B. striata*, and they exhibit a wide range of biologic and pharmacologic properties. Efficacious use of *B. striata* is dependent upon the connection between these chemical components and their specific bioactivities. Based on extensive biologic testing, numerous phytochemicals identified in *B. striata* are efficacious against one or more diseases. Developing and applying monomer compounds isolated from this herb is another development direction. Therefore, *B. striata* has great potential to be further mined for its pharmacological effects.

These studies on component detection, pharmacological probing, and clinical exploration conducted on *B. striata* have provided us with additional data for this valuable herb. Additional systematic studies will offer sufficient proof for establishing the efficacy and safety of this herb so it can be used as a medicine from a scientific point of view. We hope that this review will allow further research and development of this unique plant.

## Author Contributions

Conceptualization: DX. Project administration: DX, JC. Writing – original draft: YP. Writing – review and editing: DX, JC.

## Funding

This research was financially supported by the National Natural Science Foundation of China (31560079, 31560102, 31960074), the PhD Science Foundation of Zunyi Medical University (F-809), the Talent Growth Project of the Guizhou Education Department (KY[2017]194), and the Research Project of Guizhou Administration of Traditional Chinese Medicine (QZYY-2019-060).

## Conflict of Interest

The authors declare that the research was conducted in the absence of any commercial or financial relationships that could be construed as a potential conflict of interest.
